# *Minimising impairment*: Protocol for a multicentre randomised controlled trial of upper limb orthoses for children with cerebral palsy

**DOI:** 10.1186/s12887-016-0608-8

**Published:** 2016-05-27

**Authors:** Christine Imms, Margaret Wallen, Catherine Elliott, Brian Hoare, Melinda Randall, Susan Greaves, Brooke Adair, Elizabeth Bradshaw, Rob Carter, Francesca Orsini, Sophy T. F. Shih, Dinah Reddihough

**Affiliations:** Centre for Disability and Development Research, Faculty of Health Sciences, Australian Catholic University, 17 Young Street, Fitzroy, VIC 3065 Australia; Cerebral Palsy Alliance, PO Box 6427, Frenchs Forest, NSW 2086 Australia; School of Occupational Therapy and Social Work, Curtin University, Bentley, Australia; Monash Children’s Hospital, Clayton, Australia; Royal Children’s Hospital, Flemington Rd, Parkville, 3052 Australia; Deakin University, Building BC, Room BC3.113, 221 Burwood Highway, Burwood, Australia; Murdoch Childrens Research Institute, Parkville, Australia

**Keywords:** Upper extremity, Splint, Orthosis, Children, Cerebral palsy, Occupational therapy, Intervention, Randomised trial, Cost-effectiveness

## Abstract

**Background:**

Upper limb orthoses are frequently prescribed for children with cerebral palsy (CP) who have muscle overactivity predominantly due to spasticity, with little evidence of long-term effectiveness. Clinical consensus is that orthoses help to preserve range of movement: nevertheless, they can be complex to construct, expensive, uncomfortable and require commitment from parents and children to wear. This protocol paper describes a randomised controlled trial to evaluate whether long-term use of rigid wrist/hand orthoses (WHO) in children with CP, combined with usual multidisciplinary care, can prevent or reduce musculoskeletal impairments, including muscle stiffness/tone and loss of movement range, compared to usual multidisciplinary care alone.

**Methods/design:**

This pragmatic, multicentre, assessor-blinded randomised controlled trial with economic analysis will recruit 194 children with CP, aged 5–15 years, who present with flexor muscle stiffness of the wrist and/or fingers/thumb (Modified Ashworth Scale score ≥1). Children, recruited from treatment centres in Victoria, New South Wales and Western Australia, will be randomised to groups (1:1 allocation) using concealed procedures. All children will receive care typically provided by their treating organisation. The treatment group will receive a custom-made serially adjustable rigid WHO, prescribed for 6 h nightly (or daily) to wear for 3 years. An application developed for mobile devices will monitor WHO wearing time and adverse events. The control group will not receive a WHO, and will cease wearing one if previously prescribed. Outcomes will be measured 6 monthly over a period of 3 years. The primary outcome is passive range of wrist extension, measured with fingers extended using a goniometer at 3 years. Secondary outcomes include muscle stiffness, spasticity, pain, grip strength and hand deformity. Activity, participation, quality of life, cost and cost-effectiveness will also be assessed.

**Discussion:**

This study will provide evidence to inform clinicians, services, funding agencies and parents/carers of children with CP whether the provision of a rigid WHO to reduce upper limb impairment, in combination with usual multidisciplinary care, is worth the effort and costs.

**Trial registration:**

ANZ Clinical Trials Registry: U1111-1164-0572.

## Background

Cerebral palsy (CP), the most common physical disability in childhood, is a group of disorders of the development of movement and posture that occur as a result of disturbances in the foetal or infant brain [1]. The motor impairment may be accompanied by co-morbidities, including epilepsy, vision or hearing loss, intellectual disability, disorders of communication, behavioural difficulties, and secondary musculoskeletal problems [1]. The most common motor disorder in CP is spasticity, occurring in 86 % of individuals [2]. Spasticity is a velocity-dependent increase in the tonic stretch reflex, with exaggerated tendon reflexes [3] and is characterised by slow, effortful movement [4].

This research is embedded within an International Classification of Functioning, Disability and Health (ICF) framework [5] that articulates a dynamic interaction between impairments at body structure and function level, activity performance and participation. At the body function level, muscle over-activity as result of spasticity and/or dystonia plays a significant role in the development of secondary musculoskeletal impairments in the upper limbs [6] that are common in CP. Secondary impairments include muscle stiffness, loss of active range of movement, joint contracture and pain. Diminished skeletal muscle growth is a key feature in the aetiology of contracture and deformity [6]. Persistent over-activity of skeletal muscle, and subsequent maintenance of a shortened position, can cause a failure of longitudinal muscle growth and muscle adaptation, including increased resistance to passive stretch or *stiffness* [6, 7]. Subsequently there is a biomechanical imbalance of bone to muscle, as bone continues to grow and muscle growth is impeded [7]. The combined impact of these factors can result in soft tissue retraction, loss of active and passive range of motion and joint contracture [6, 8].

Progressive changes in muscle length and stiffening of joints in the upper limbs can ultimately result in a limited ability to reach, grasp and manipulate objects or, in some individuals, a complete lack of functional use of the hands. Strong correlations exist between the degree of upper limb deformity and activity performance [9]. When combined with neurological dysfunction, upper-limb musculoskeletal impairments significantly impact on the ability of children to use their hands to perform daily activities, attain age appropriate independence and develop the autonomy and skills required to participate in activities of importance in home, school and community environments [10, 11].

Children with CP are not born with musculoskeletal impairments. There is evidence however, that in children with spastic motor types, these impairments begin to manifest prior to three years of age [12] and that increasing stiffness and progressive loss of range of movement occurs throughout childhood and adolescence [13–15].

A range of treatment options are available for children with CP that specifically focus on improving hand use. These include activity-based interventions such as goal-directed training, intensive bimanual therapy, modified constraint-induced movement therapy and home programs. Each of these interventions aim to achieve child/parent-focused goals, and has high-level evidence supporting their effectiveness for increasing activity-level performance and goal achievement [16]. Little evidence is available however, about whether activity-level interventions improve range of movement and reduce secondary musculoskeletal impairments. In addition to activity-based therapies, injection of Botulinum toxin A (BoNT-A) into overactive muscle groups is known to reduce muscle overactivity and has been associated with improved range of movement during a period of chemical denervation, therefore enhancing the effects of upper extremity therapy and the potential for goal achievement and activity performance [17]. Nevertheless, BoNT-A alone in the upper limbs has been shown to have little sustained effect on range of movement [17]. Upper limb surgery is also available to correct deformity once present, although outcomes are variable [18].

Removable orthoses (also called splints) are applied to the forearms, wrists and hands with the goal of either maintaining muscle length and joint range of movement through sustained stretch, or enhancing functional performance. Although orthoses are commonly integrated into intervention strategies with children with CP, there is little evidence supporting the use of upper limb orthoses and wide variation in their prescription, manufacture and intended aims [10, 19]. One controlled trial [20] demonstrated improved effect of BoNT-A when combined with static splinting (term used in the trial by Kanellopoulos et al.) in children with CP. A Cochrane systematic review by Katalinic et al. [21], which included adults and children with a broad range of neurological and non-neurological conditions, demonstrated little benefit of stretching for preventing or reducing contractures. The review concluded that the use of interventions that provided a stretch to muscles, such as upper limb orthoses, be ceased [21]. The application of these findings to children with CP however, is limited. Only five of the 35 randomised trials included children. Of these, three studies included children with CP and each of these evaluated the effects of casting (one in the upper and two in the lower limb) as opposed to orthosis wear. A more recent systematic review of the effectiveness of upper limb orthoses for children with CP found more equivocal evidence than Katalinic et al. and recommended further methodologically strong research be completed to more effectively inform practice [22].

The clinical rationale for providing upper extremity orthoses is multi-faceted, with both short and long term goals. The focus of the current study is on rigid wrist/hand orthoses (WHO). The primary goal of wearing rigid WHO for all children with CP is to prevent the development of muscle stiffness, maintain the integrity of soft tissues and prevent the development of abnormal postures and long-term deformity. Due to the diverse nature of CP, the secondary goals generally depend on the child’s Manual Ability Classification System (MACS) level. The MACS is a five-level system that describes how children use their hands to handle objects during daily activities [23]. For children in MACS levels I to III, who are able to handle objects in daily life, the secondary aims of WHO prescription are to improve or maintain activity performance and participation through maintenance of good posture for functional use of the hand. Children in MACS levels IV and V have little or no functional use of the upper limb(s) and the aim of wearing of a rigid WHO is to maintain posture to facilitate ease of care-giving during daily activities such as bathing, dressing and positioning. Wearing of a rigid WHO is also used to prevent complications associated with muscle shortening such as pain and poor palmar skin hygiene.

Application of the results of previous research has been limited by the length of time in which WHO have been applied and evaluated (often <6 months). These time frames are often dictated by the cost of implementing a trial. However, orthosis wear is an intervention aiming for long-term benefits (i.e. the reduction of contracture over time), thus clinicians are appropriately reluctant to change practice based on short-term research evidence. A well-designed large trial with a long intervention and follow-up period is now critical to determine the long-term outcome of this intervention. This 3 year trial has been designed to balance the need for longitudinal evidence with the complexities of attaining prolonged adherence of participants to an intervention within a controlled trial.

Effective and feasible rigid WHO are those where the benefits outweigh the risks associated with the intervention such as client discomfort, potential for skin breakdown, carer burden in maintaining routine application, follow-up appointments for manufacture and adjustment. Provision of WHO should also contribute positively to the long-term goals of children and families in terms of achievement of participation in meaningful activities during childhood and/or improved ease of caring for children with CP. Cost-effective rigid WHO are those where the benefits outweigh the net costs (defined as cost of the intervention minus the cost offsets) and/or where the relationship between the net costs and outcomes is deemed acceptable (i.e. less than a common decision threshold in Australia, such as <$50,000 per Quality Adjusted Life Year (QALY)). Affordable rigid WHO are those where the financial costs for materials, construction and monitoring are within the available budget of third party funders and/or parents.

Wrist-hand orthoses interventions, in combination with activity-based therapy, are aimed at maintaining muscle length, strength and balance, which are required for optimum force generation, effective grasp and manipulation [24] and therefore functional use of affected hands in daily activities. The primary aim of this research is, therefore, to evaluate whether use of rigid WHO over 3 years in children with CP, combined with usual multidisciplinary care, can prevent or reduce musculoskeletal impairment including loss of range of movement and muscle stiffness at the wrist, compared to usual multidisciplinary care alone. The impact of WHO wear on pain, activity performance and participation, as well as ease of caregiving for families will be evaluated along with an assessment of cost-effectiveness of the intervention.

## Research questions

In children aged 5 to 15 years with CP, does the provision of a serially adjustable rigid WHO in combination with usual multidisciplinary care, compared to usual multidisciplinary care alone:

1. ***Reduce*** body function and structure impairment including contracture, defined as fixed loss of range of movement, ***or prevent*** further loss of range of movement at the wrist, and reduce pain and muscle stiffness at three years from the beginning of the study? This is the primary research question.
2. ***Improve*** activity performance and/or ease of caregiving at three years from the beginning of the study?
3. Influence participation and quality of life at three years from the beginning of the study?

In children aged ≥5 years with CP:

4. What are the effects of the interaction between age and provision of the WHO plus usual care, and the interaction between severity and provision of the WHO plus usual care, compared to usual care alone, in reducing or preventing further loss of range of movement, muscle stiffness or pain, or improving activity performance or ease of caregiving, at three years from the beginning of the study?
5. What are the incremental costs and potential cost offsets of adding a rigid WHO to usual care compared with usual care alone?
6. From a health sector perspective, what is the cost-effectiveness of providing a rigid WHO combined with usual care, compared to usual care alone?

### Trial registration

This study has been registered with the Australian and New Zealand Clinical Trials Registry: U1111-1164-0572. Table 1 displays key registration data.

**Table 1:** Minimising impairment trial registration data: Protocol Version 3: 30.10.2014

Data Category	Information
Primary registry and trial identification number	ANZ Clinical Trials Register: U1111-1164-0572
Date of registration in primary registry	5.12.2014
Secondary identification numbers	N/A
Sources of money or material support	Australian Catholic University; National Health and Medical Research Council, Australia
Primary Sponsor	Investigator led: Professor Christine Imms
Contact for public queries	Dr Melinda Randall: Melinda.randall@acu.edu.au
Contact for scientific queries	Prof Christine Imms: Christine.imms@acu.edu.au
Public title	*Minimising impairment*: a multicentre randomised controlled trial of upper limb orthoses for children with cerebral palsy.
Scientific title	Does wearing a rigid upper limb wrist hand orthosis in combination with evidence informed occupational therapy, compared to evidence informed occupational therapy alone, reduce wrist/hand impairment and improve activity and participation outcomes in children aged 5–15 years with cerebral palsy?
Countries of recruitment	Australia
Health condition studied	Cerebral palsy
Intervention	Intervention: custom-made serially adjustable rigid wrist hand orthoses to maintain the flexor compartment (muscles of the wrist, fingers and thumb) in a lengthened position to avoid shortening of the musculo-tendinous unit and other soft tissue.
	Control: The control group will not receive a rigid wrist/hand orthosis.
	Both groups: will receive care typically provided by their usual treating organisation. Possible treatments may include developmentally appropriate, goal focused and evidence-informed occupational therapy, the use of equipment or BoNT-A injections.
Key inclusion/exclusion criteria	Ages eligible for study: 5–15 years; Gender eligible: both;
	Inclusion criteria: A confirmed diagnosis of cerebral palsy as recorded in the medical history; Presence of flexor muscle stiffness - score at least 1 on the Modified Ashworth Scale during wrist extension with fingers extended; May or may not already exhibit contracture at the wrist.
	Exclusion criteria: upper limb dystonia without the presence of spasticity; an allergy or sensitivity to the materials used to construct orthoses; if families are unable to access the study site at the necessary times; and if families identify factors (e.g. child’s behaviour) that impact significantly on their ability to carry out the intervention
Study type	Interventional
	Allocation: randomised; intervention model: parallel assignment; Masking: Single blind.
	Primary purpose:
Date of first enrolment	28.8.2015
Target sample size	194
Recruitment status	Recruiting
Primary outcomes	Passive range of wrist extension (measured with the fingers extended) measured using a goniometer at 3 years; Active range of wrist movement measured using standardised goniometric measurement and use of clinometer for measures of supination and inertial motion sensors measures at 3 years;
Key secondary outcomes	Body function outcomes: Muscle tone; muscle spasticity; grip strength; hand deformity and pain.
	Activity outcomes: Self-care; Manual ability; Hand speed and dexterity; ease of care.
	Participation outcomes: Attendance and involvement in home, school and community activities.
	Quality of Life outcomes: Child and parent.
	Health economics outcomes: relative cost and cost-effectiveness.

## Methods

### Design

This study is a pragmatic, prospective, multicentre, assessor-blinded randomised controlled trial (RCT) with economic analysis that aims to measure the effectiveness of the intervention under usual multidisciplinary care conditions [25]. It will incorporate a two-arm, parallel group superiority design and 1:1 allocation of children to wear WHO and participate in usual multi-disciplinary care or to multidisciplinary care alone. Assessments will be completed at baseline, and every 6 months for 3 years.

### Participants

#### Child participants

Eligible children will be aged 5 to 15 years at the time of recruitment; diagnosed with CP as recorded in their medical history; and present with flexor muscle stiffness of the wrist as indicated by a Modified Ashworth Scale score ≥1 during wrist extension with fingers extended. Children may or may not already exhibit contracture. Parents are required to be able to understand written and spoken English as it is not feasible to translate all study materials into languages other than English.

Prior upper limb surgery is not an exclusion criteria, however, to avoid interfering with surgical outcomes children will only be eligible if they are at least 12 months post-surgery. In addition, children will be excluded from this study if they have upper limb dystonia without the presence of spasticity; an allergy or sensitivity to the materials used to construct orthoses; if families are unable to access the study site at the necessary times; and if families identify factors (e.g. child’s behaviour) that impact significantly on their ability to carry out the intervention.

#### Therapist participants

Therapists providing the WHO for any child recruited to the study may be occupational therapists or physiotherapists. They will be asked to consent to providing data related to their discipline and experience, and about the design and fabrication of orthoses they provide to recruited child-participants at each occasion of manufacture or adjustment.

### Ethics

Ethical approval has been received from the Australian Catholic University (HREC: 2014 317 V), Cerebral Palsy Alliance in NSW (HREC: 214-08-02), Monash Children’s Hospital (HREC: 14199B) and The Royal Children’s Hospital (HREC: 34280A) in Victoria and the Princess Margaret Hospital in Western Australia (HREC: 2014060). Parents or guardians of all child-participants will provide informed written consent for their child to take part in the study, as well as consent to complete questionnaires. Children aged 12 years or older will be asked to assent to taking part in the trial if they are capable of doing so. Modifications to the protocol will be reported to each HREC, all investigators and noted on the trial registry.

### Sample size

Sample size calculations were performed according to the primary dependent variable: passive range of movement of wrist extension (measured with fingers extended) at 3 years. Sample size calculations were based on data provided by Rameckers et al. [26] who reported a baseline standard deviation (SD) of 12.4° of passive wrist extension in a group with hemiplegic CP. Rameckers et al. [26] included a more homogeneous group of children than we intend and, therefore, in the absence of clear evidence in the literature, the variability has been estimated to approximate 22° (10° greater than Rameckers et al.’s more homogenous group). Based on a SD of 22° for the primary outcome, 77 participants per group would be required to detect a 10° between-group difference in passive range of movement at 36 months with 80 % power and a two-tailed level of significance of 0.05. A 10° difference was chosen as differences greater than 5 to 10° of movement are deemed clinically important [21]. We used data from our previous successfully completed RCTs of upper limb interventions for children with CP to estimate withdrawal and loss-to-follow-up rates. Prior withdrawal rates ranged from 0 to 10 %, with an additional loss-to-follow up in these RCTs ranging from 0 to 3 % over periods of 6 months. We anticipate that loss-to-follow up in this 3 year study will be higher than 3 %, and thus estimate a 10 % loss. Consequently we predict a combined withdrawal/loss-to-follow-up rate of 20 %. Based on these estimates, we aim to recruit 194 children. Of note, children may have both limbs included in the study. The presence of two limbs was not included in the sample estimates but will be used in the analyses. Including both limbs will increase study power; hence the sample size is expected to be conservative if two limbs are included for some children. Randomised trial experience in Australia suggests we will require at least 12 months to recruit the required sample.

### Recruitment

**Fig. 1:**
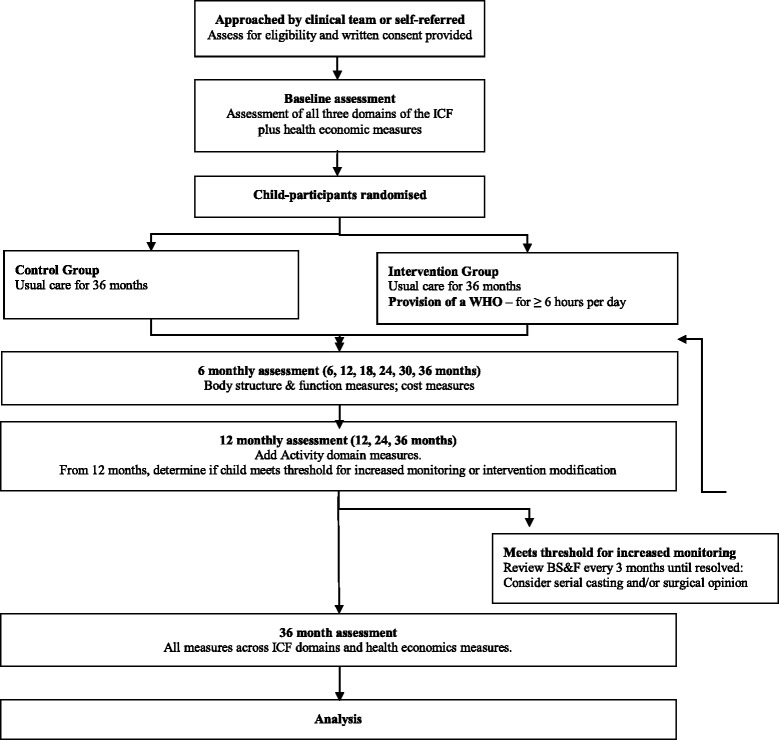
Study flow diagram. ICF: International Classification of Functioning, Disability and Health; BS&F: body structure and function

This multicentre trial will be conducted in Victoria (Monash Children’s Hospital and The Royal Children’s Hospital), Western Australia (Princess Margaret Hospital and The Ability Centre) and New South Wales (Cerebral Palsy Alliance). The treating clinical teams at each trial site will identify potential participants and provide written and verbal information about the study to potential families (see Fig. 1: Study flowchart). Study advertisements, which will include contact details for the project coordinator and the research assistant assigned to respective sites, will be distributed to clinicians and families. Further advertising will be done through site-specific newsletters, websites and social media with an invitation to contact study personnel or treating therapists for more information about the study. Eligibility will be determined through discussion with parents and clinical examination of the child’s upper limb/s.

### Randomisation

Once consent and assent (for those children aged 12 years or more) has been obtained by the research assistant, and following baseline assessment, children will be randomised to either the treatment or comparison group with an allocation ratio of 1:1, using a web-based randomisation procedure to ensure concealed allocation. Randomisation will be completed using randomly permuted blocks of variable length, stratified by study centre (5 strata), and by passive wrist range of movement (3 strata: wrist extension >0° (fingers extended); wrist extension between −45°-0° (fingers extended); or wrist extension < -45° (with or without finger extension)). For children with bilateral involvement, where both limbs meet inclusion criteria, both limbs will be allocated to the same intervention based on the randomisation sequence and stratification will be based on the passive wrist range of the more involved limb. Data will be collected for both upper limbs and included in the analysis at the end of the study for limb-based outcomes.

### Interventions

All children will receive care typically provided by their usual treating organisation. Possible treatments include occupational therapy (e.g. goal-directed training, intensive bimanual therapy), prescription and use of equipment and /or BoNT-A injections. Some children may already have been prescribed a WHO. The WHO of those children allocated to the intervention group will be assessed to ensure it meets the intervention protocol, and adjusted if necessary. Those in the control will cease wearing their WHO. Due to the pragmatic nature of this trial, study duration and anticipated heterogeneity of the study sample and possible co-interventions, it will not be possible to standardise co-interventions, however a detailed log will be kept and efficacy/efficiency implications of a non-standardised comparator will be carefully assessed.

#### Treatment group

The treatment group will receive a custom-made serially adjustable rigid WHO to maintain the flexor compartment (muscles of the wrist, fingers and thumb) in a lengthened position to avoid shortening of the musculo-tendinous unit and other soft tissue. For children with unilateral involvement only one hand will be provided with an orthosis. For children with bilateral involvement, both hands will be treated if clinically indicated. The WHO may involve variations of a volar or dorsal/volar orthosis. Volar orthoses are placed on the palmar aspect of the hand and forearm; dorsal/volar orthoses have a component on the palm and a second, linked component on the back of the forearm. Both types of orthoses are designed and fabricated to achieve the same aims. Orthoses will be made of thermoplastic material, which when gently heated loses rigidity allowing it to be conformed to the child’s arm in the required position. On cooling, thermoplastics regain rigidity. Straps will be used to maintain the correct position of the child’s wrist/hand in the orthosis.

As there is no evidence to guide the optimal position to achieve with orthoses, wearing regimes or follow-up procedures, a national group of expert occupational therapists developed consensus-based guidelines to include in the study operating procedures. Rather than specify muscle and joint positions to achieve with orthoses, these guidelines provide principles to guide individualised positioning of the thumb, fingers and wrist in the orthosis based on the available passive range of motion of the flexor compartment and the relative contribution of wrist and finger flexor stiffness. Briefly, the thumb is positioned midway between full extension and abduction to maintain web space while avoiding metacarpal-phalangeal (MCP) joint hyperextension. Fingers are placed in a small amount of flexion (10–30° at MCP and inter-phalangeal joints) to prevent the hand slipping in the orthosis. The orthosis is then fabricated to maintain thumb and finger position with the wrist positioned in the orthosis to achieve stretch to the flexor compartment. Each WHO will be fabricated following a clinical assessment of the child’s range of motion and muscle tone. Therapists in participating study sites will fabricate the orthoses as part of their usual clinical duties to achieve an individualised position of the wrist and hand according to pre-determined principles outlined in the consensus-based guidelines.

To achieve a prolonged positioning effect, children will be asked to wear the orthosis for a minimum of 6 h for each 24 h period of the 3 years of the study [27]. Although night time wear is recommended, it may be appropriate for some children to wear the orthosis during the day: for instance, children with more severe impairments who do not use their hands to carry out activities and therefore for whom wearing a WHO is unlikely to impact on functional hand use. Children with bilateral upper limb involvement where both hands meet the criteria for wearing an orthosis will be asked to wear both orthoses each night. If sleep quality is compromised as a result of concurrently worn bilateral orthoses, recommendations will be to: (i) wear both orthoses each day; or (ii) wear one orthosis at night, the other during the day; or (iii) alternate wear of each orthosis on alternating nights.

The therapists who manufacture the WHO will monitor them in a manner consistent with usual clinical practice. Orthoses will be replaced or serially adjusted over time according to clinical indications to accommodate growth, ensure good fit and maintain appropriate positioning. Serial adjustment involves re-heating the orthosis and remoulding it to achieve an adjusted position. The altered position may be in a position of greater wrist/finger extension in response to improvement in range of motion or reduced muscle stiffness, or in a position of reduced wrist/finger position if range of motion decreases or muscle stiffness increases.

Families will be provided with verbal and written instructions for donning, caring for and cleaning the orthoses; wearing regimes and guidance for facilitating the child to wear orthoses. Consistent with usual clinical practice, families will be asked to contact the therapist who manufactured the WHO if any difficulties with orthosis wear arise such as rash, red marks, bruises, pain or difficulties wearing the orthosis for 6 h per 24 h period.

Fidelity of intervention will be considered in three ways. Therapists fabricating orthoses will be educated by study personnel regarding study requirements, orthoses fabrication and wearing principles, which will also be documented in the standard operating procedures provided to each participating therapist. Participating therapists will complete a questionnaire to record details of the design and fabrication of the orthosis and photograph each orthosis on completion of fabrication to provide study personnel with a digital image for evaluating orthosis manufacture against the pre-determined principles. These photographs will also enable assessment of the nature and design of orthoses used in the study. Data related to adjustments made to the WHO over the study period will also be collected. Finally, the frequency and duration that each child wears the orthosis will be recorded daily using a custom-designed application for Android and Apple mobile devices known as Therapy App (TherApp) (see *Intervention adherence* for details).

#### Control group

The control group will receive usual multidisciplinary care without a WHO. Prior to consenting, parents will understand that a child entering the study with a prescribed WHO and allocated to the control group will cease wearing it. Parents and children will be advised there is debate about effectiveness; the study will generate evidence to inform future practice. They will also be informed that children in either group could lose ROM during the trial period as this is the natural history of CP, and because of this, their child will be monitored closely throughout the 3 year study period.

#### Intervention – modifications

Children in either group could lose range of movement through the trial period as this is the natural history of CP. In addition, the trial will run for an extended period (3 years) during which growth and physical development will influence children’s outcomes. Both groups will, therefore, be carefully monitored for loss of range of motion over time.

Children will be monitored for the first 12 months following enrolment according to the study protocol and will not receive any modification to the intervention during this period. Children assessed at or after the 12-month time point who have lost ≥30° passive range of wrist extension or increased by ≥2 categories on the Modified Ashworth Scale, compared to baseline, in either group will be eligible for increased monitoring. This involves reassessment at 3-monthly intervals and may, in consultation with the child’s parents and treating team, involve either serial casting to regain range of movement and/or a referral for a surgical opinion. Casting is common clinical practice in children with CP to achieve increased passive range of motion over the short term [10].

#### Criteria for withdrawal

Withdrawal criteria, which apply to all children, have been specified *a-priori* to ensure child safety, quality of study data, and the ability to answer the research questions. Children who receive a diagnosis other than CP or their movement disorder evolves to dystonia without the presence of spasticity, will be withdrawn from the study and all data removed from analyses. Children who have a surgical intervention to the included upper limb or more than three episodes of casting as a modification to treatment during the course of the study may discontinue study treatment but will be followed until study completion and be included in the statistical analyses where possible.

#### Concomitant care

Children may participate in a range of upper limb interventions throughout the study according to usual multidisciplinary care, such as assistive technology or equipment and devices, upper limb BoNT-A injections, intensive bimanual therapy, early intervention, goal-directed training, home programs, modified constraint-induced movement therapy, or parent education/coaching [16]. Choice of concomitant interventions will be determined by the child’s family and treating team and will be recorded.

#### Intervention adherence

Adherence to the allocated intervention will be recorded using TherApp, which is the equivalent to an electronic diary. TherApp will be used in conjunction with a study-specific questionnaire to assist with measurement of WHO adherence, type of co-interventions and incidence of side effects from orthosis wear. Families of children with CP were consulted about the content, feasibility and implementation of TherApp.

Group allocation (concealed from the assessors) and personal details will be programmed into TherApp at the time of randomisation to ensure appropriate questions are asked about each child. Parents nominate the time for a daily and/or weekly reminder to be sent to their phone or device. For children in the intervention group, TherApp sends a daily prompt to parents requesting information on daily wearing duration and a weekly request for details on adverse events due to wearing orthoses, or any difficulties the child or family experience with wearing orthoses. TherApp will prompt parents to contact their child’s treating therapist if needed. We anticipate this will facilitate adherence to the provision of the WHO and contribute to treatment fidelity. If TherApp registers no response to the daily prompt, an automated email alert to the research assistant enables appropriate follow up. Parents of children in both groups will also receive a weekly TherApp request to record upper limb therapy received in the previous week. Data recorded on TherApp will be extracted prior to each assessment period and supplemented with 6-monthly parent interviews to record additional details about concomitant therapy in the preceding period.

In addition to the TherApp, a novel pressure sensor has been engineered to record the time orthoses are worn. Three sensors will be implanted in the contact-surface of the orthosis (under the thumb, fingers and wrist). The sensors developed for this study use wireless, blue tooth technology and have a sampling rate of 1Hz per second. This tiny device is printed on material safe for human consumption using 3-Dimensional printing technology to meet the combined requirements of accurate measurement, protection of skin integrity associated with device pressure and protection of the child from swallowing a small item. Data obtained from the sensor will be used to validate parent-report data extracted from the TherApp in a subset of participants.

## Data collection and analyses

### Assessors and details of blinding

Assessors will be occupational therapists or physiotherapists blinded to the treatment group of the child. Each assessor will be trained by a chief investigator in reliable administration of all measures and provided with a study assessment protocol to ensure consistency of assessment techniques between assessors. Multiple efforts will be made to retain blinded status of assessors. Treating therapists, research personnel, child-participants and families will be routinely reminded that it is critical that blinding of the assessor is retained and the assessor will remind the family of this on initial contact at each assessment. Study numbers allocated to children will not contain a fixed code denoting intervention group. The success of blinding will not be measured as methodological experts argue against such practice [28]. Therapists providing the WHO are unable to be blinded to treatment group and hence may adjust co-interventions differently in the two groups. Measurement of co-interventions and comparisons between groups is therefore is an important part of this trial. Blinding of parents and participants to intervention group is not possible.

### Demographic and diagnostic characteristics

Consistent with best practice in CP [1], the severity of CP will be assessed and classified at baseline using the Gross Motor Function Classification System [29], Manual Ability Classification System [23], Communication Function Classification System [30] and Bimanual Fine Motor Function scale [31]. In addition, the type of movement disorder (that is, spastic or mixed) and severity of spasticity in the included upper limb(s) will be rated using the Hypertonia Assessment Tool [32] and the Australian Spasticity Assessment Scale [33, 34] respectively. Information from these tools will help to describe the characteristics of the study sample and be used in post-hoc analyses of outcomes. A study specific questionnaire will measure a range of demographic and other variables including age, gender, associated conditions (intellectual disability, sensory impairments), family configuration, range of services received and socio-economic status as defined using the Socio-Economic Index for Areas data [35].

### Outcome measures

The primary outcome measure is passive range of wrist extension (measured with the fingers extended). Range of wrist movement is operationalised from -70° (full wrist flexion) to + 80° (full wrist extension) where 0° indicates a neutral position. Range of movement will be measured using a goniometer for extension/flexion movements, an inclinometer for supination/pronation and inertial motion sensors. Inertial motion sensors, constructed specifically for the trial, will be used to measure active and passive wrist extension, with fingers extended as well as functional range of wrist extension elicited during standardised tasks. Other measures across the domains of the ICF will also be used to evaluate the effect of the intervention. Table 2 displays each variable, the measurement tool selected and provides an overview of psychometric evidence for the selected tools.

**Table 2:** Variables and outcome measures

Variable	Measurement tool	Additional information
ICF level: Body function: baseline, 6, 12, 18, 24, 30, 36 months		
Passive range of motion: elbow extension, wrist extension (with fingers extended), wrist extension (with fingers flexed), supination	Standardised goniometric measurement; inclinometer for measures of supination;	Goniometric measurements have a high level of intra-rater reliability when measuring passive range of movement in the lower limb in children with CP (ICC >.80) and SEM of 3.5° [44, 45].
	Inertial Motion sensors.	Inertial motion sensors (see additional information below) will be used to measure passive wrist extension with fingers extended only.
Active range of movement: elbow extension, wrist extension (with fingers extended), wrist extension (with fingers flexed), supination	Standardised goniometric measurement and use of inclinometer for measures of supination.	See additional information above.
	Inertial Motion sensors	Inertial motion sensors (see additional information below) will be used to measure active wrist extension with fingers extended only.
Functional range of wrist extension during standardised tasks.	Inertial Motion sensors.	A wireless inertial motion sensor for children has been designed and engineered for this trial to measure wrist flexion/extension movement during functional activity. The sensors use a combination of inertial sensor technologies to provide an accurate estimate of orientation referenced to a fixed frame [46]. Once correctly positioned they wirelessly capture movement with 3° of freedom in a virtual reality environment to provide continuous kinematic data during unrestricted functional movements. The validity and reliability of the newly developed sensor has been assessed with 10 children with CP (aged 4–12 years) against 3DMA, the ‘gold standard’ method to quantify movement. Preliminary data demonstrates the inertial motion sensors have excellent static and dynamic accuracy (+/-0.5 and +/-1.2° respectively).
Muscle stiffness (finger flexors, wrist flexors, pronators and elbow flexors)	Modified Ashworth Scale [47]	The six point Modified Ashworth Scale has moderate intra-rater reliability when assessing the elbow (ICC 0.66) and wrist flexors (ICC 0.57) in children with CP [48].
Muscle spasticity (finger flexors, wrist flexors, pronators and elbow flexors)	Modified Tardieu Scale [47]	The Modified Tardieu Scale has moderate to high intra-rater reliability when assessing the elbow (ICC 0.65) and wrist flexors (ICC 0.92) in children with CP [48].
	Australian Spasticity Scale [33]	The Australian Spasticity Assessment Scale has demonstrated moderate to high inter-rater agreement (47–100 %) [33]
Grip strength	Hand held dynamometer (CITEC)	Dynamometery has been shown to have excellent levels of inter-rater (ICC 0.95) and test-re-test reliability (ICC 0.96) when measuring strength in the hand of children with hemiplegic CP [49].
Hand deformity	Neurological Hand Deformity Classification Scale [50]	The Neurological Hand Deformity Classification has evidence of reliability for children with spastic cerebral palsy with high inter-rater agreement (Kappa 0.87) and intra-rater agreement (Kappa 0.91) [15]
Thumb position	House Thumb in Palm classification [51]	This measure has been developed for children with CP based on the predictors of surgical success and has been found to be reliable: Kappa = 0.73 (rater agreement) and 0.74 (test-re-test agreement) [49, 52].
Hand pain	Study specific questionnaire	The study specific questionnaire was developed for this study to document parent perception of domains unable to be captured in existing measures. Questions will be completed by the child where possible or by a parent/carer proxy. Although proxy respondents are known to underestimate pain, parent-reported pain will be required for children who are more severely cognitively impaired or unable to communicate their pain effectively.
Activity domain of the ICF: baseline, 12, 24 & 36 months		
Self-care skills	Pediatric Evaluation of Disability Inventory – Computer Adaptive Test [53]	This is a standardised assessment of how children with impairments function in the context of their daily life. The Pediatric Evaluation of Disability- Computer Aided Test provides an accurate and precise assessment of abilities in four functional domains (ICC 0.99). For this trial only data from the Daily Activities domain will be collected.
Manual ability	ABILHAND-Kids [54]	This tool has been Rasch analysed and has demonstrated validity and appropriate range and measurement precision for clinical practice and research: reliability: *R* = 0.94; reproducibility over time: *R* = 0.91 [54].
Speed and dexterity	Box and Blocks Test [55]	This test has a high level of intra-rater (ICC 0.99) and test-retest reliability (ICC 0.85) [56].
Hand function	Modified House Scale [57]	This scale is reliable in children with CP: inter rater reliability (ICC 0.94-0.96); intra rater reliability (ICC 0.93-0.96) [57]. Rasch analysis was performed on the original scale and the items reduced: analysis suggests that the modified version demonstrates good construct validity [58].
Ease of care-giving	Study specific questionnaire	Parent response to specific questions regarding the child’s ability to use their hands in self-care tasks or, for children with severe forms of cerebral palsy the ease with which parents or carer’s can complete daily tasks of care for them.
Participation domain of ICF: Baseline & 3 years only		
Participation	Participation and Environment Measure-Child & Youth [59]	Designed to measure frequency of participation, involvement during participation and the impact of the environment on participation in children aged 5 to17 years [59]. This measure captures participation outcomes in home, school and community contexts. Reliability of the frequency scales (ICC range 0.58-0.84) and involvement scales (ICC 0.69-0.76 is moderate to high [59].
Child Health related quality of life and care-giving burden	Cerebral Palsy Quality of Life Questionnaire – Child and Teen versions [60, 61]	Due to the varying ages and abilities of the child-participants, both parent- and self-report versions of the Child or Teen CP Quality of Life will be used to measure quality of life. Test-re-test reliability for the Child version was high (ranged from ICC 0.76 to 0.89 across 7 scales) [61], and moderate to high for the Teen version (ICC 0.57 to 0.88) [60]
Health economic measures: Baseline, 12, 24, 36 months		
Cost Effectiveness Analysis (CEA)	Study specific questionnaire	Data on type and number of health professional appointments attended by child in preceding 6-month time period will be utilised for calculation of healthcare cost as well as out of pocket costs to families. Net incremental costs expressed as ICER to meaningful clinical and physical outcomes (e.g. selected from body function domains; activity domains; and the clinical quality of life questionnaire).
Cost Utility Analysis (CUA)	Child Health Utility -9 Dimensions [37]	Net ICER to the quality of life improvement for children and parents/carers expressed as QALY using an economic MAUI. Where possible the Child Health Utility will be completed along with the parent proxy version. The Child Health Utility has 9 items, takes 2-3 min to complete and covers worry, sadness, pain, tiredness, annoyance, school work, sleep, daily routine and ability to join in activities. The Child Health Utility-9D demonstrated good validity and high levels of agreement with a similar instrument (ICC: 0.742) [62]. The parent measure of quality of life, the Assessment of Quality of Life 8 Dimensions has high reliability (ICC 0.89) [36].
	Assessment of Quality of Life 8 Dimensions [36]	
Cost Consequences Analysis (CCA)		CEA/CUA reported alongside a broader documentation of child & family relevant outcomes

Note: *ICC* intraclass correlation coefficient, *SEM* standard error of measurement, *3DMA* three dimensional motion analysis, *ICER* Incremental Costs Effectiveness Ratio, *MAUI* multi-attribute health utility instruments, *QALY* quality adjusted life year, *CEA* Cost effectiveness analysis, *CUA* Cost utility analysis

### Economic analysis

Economic analysis in the context of trials is designed to answer one or both of two questions: i) does the treatment being evaluated offer value-for-money (i.e. ‘allocative efficiency’); and ii) if so, how best to design/implement it (i.e. ‘technical efficiency’). In this trial we are focussed on technical efficiency. Specifically, is the care pathway more cost-effective with the addition of a WHO for children with CP than without? Economic methods have been chosen therefore to focus on appraisal using trial-based data (with limited economic modelling) and to deal with variability in usual multidisciplinary care. A comprehensive analysis of usual care activities will enable: i) specification of a weighted average usual care pathway (i.e. weighted by activity prevalence); ii) incremental cost-effectiveness ratios (ICERs) presented by state/site, as well as by overall trial results; and iii) extensive sensitivity/uncertainty analyses to detail cost and outcome drivers.

The technical efficiency focus will make cost-effectiveness analysis (CEA) and cost consequences analysis (CCA) the primary analysis, with ICERs that focus on body function, activity measures and child/family quality of life and health utility outcomes from both health sector and service funder perspectives. The key trial-based ICERs are: i) the ‘net cost per 10° improvement in passive range of movement at 3 years’; ii) the ‘net cost per unit of improvement on the *Cerebral Palsy-Quality of Life’* measure; iii) the ‘net cost per *Adult Quality of Life-8 Dimensions’* [36] improvement for parent/carer; and iv) the ‘net cost per *Child Health Utility 9D’* [37] improvements for children. While economic quality of life instruments are usually focussed on value-for-money comparisons, the difficulties in modelling longer term outcomes in this trial, leads to their primary purpose being to help establish technical efficiency. ICERs will be reported as both point and range estimates. In the CCA, ICERs will be reported and interpreted alongside the full range of body function and activity measures collected. A Cost-Utility Analysis (CUA) with variable time horizons and best available data will be included in sensitivity analysis against a specified decision threshold (i.e. < $50,000 per QALY). In addition to the CEA/CCA and CUA, a broader economic approach will be undertaken to capture policy and implementation/policy issues (e.g. acceptability to stakeholders, equity impacts, feasibility of implementation, quality of the evidence base) using ***A***ssessment of ***C***ost ***E***ffectiveness (*ACE*) methods. *ACE* has been used across a series of commissioned and NHMRC-funded projects [38].

Costs will be calculated using pathway analysis to document treatment activity, specify unit prices and estimate costs and potential cost offsets across the study groups. For usual multidisciplinary care, a number of pathways will be constructed and analysed separately as well as a weighted average comparator. Costs associated with the WHO will be assessed by expenditure category (i.e. salaries, overheads, consumables) with economic data collected using a logbook; all other healthcare costs will be assessed by incidence category (i.e. who bears the cost) using available information from sources such as the Medical Benefit Schedule and Pharmaceutical Benefit Scheme. Sensitivity/uncertainty analyses will be undertaken to investigate the robustness of the ICERs to variations in key cost, pathway and outcome parameters in the trial and across sites.

### Data collection methods for child-participants who exit prematurely

Children may exit prematurely from the study because of voluntary withdrawal or termination of WHO intervention due to harm (e.g. allergic reaction to materials). Participant retention will be supported within the study through provision of routine follow-up and regular feedback on child progress via the TherApp summary report that can be generated by parents throughout the trial. Where possible, all children will be followed to the study end point (3 years) so that data are available for analyses. Lack of adherence to the treatment plan will be recorded using TherApp and will not constitute a reason for withdrawal. Reasons for withdrawal from the intervention, or the study, will be recorded to assist with management of missing data and interpretation of results.

### Monitoring of harm and adverse events

No harm or adverse events from orthoses are reported in the literature but are occasionally noted in clinical practice; these are temporary and non-sentinel. Harm arising from the WHO could include the development of pressure areas on the skin, pain, disturbed sleep or behaviour, and skin allergies from specific splint materials while wearing the orthosis, and heat during orthosis fabrication. Children in both groups are at risk of a reduction in joint range of movement as part of the natural course of CP during growth and development. Adverse events unrelated to the study may also occur and will be adjudicated by the Data Monitoring Committee. Data related to harm and/or adverse events for all children will be collected throughout the study by the therapist who manufactures the WHO (routine follow up), retrospectively by study research assistants (6 monthly) and via TherApp alerts. If the TherApp registers an adverse event an automated email alert to the study research assistant will enable appropriate follow up.

### Data management

Data will be collected using a combination of paper-based and web-based data forms supported by the secure Research Electronic Data capture (REDCap) data management system [39] hosted at the Murdoch Childrens Research Institute. REDCap supports quality control measures including rule-based data entry to reduce data entry errors. In addition, data cleaning will be undertaken. Secure electronic data storage will be undertaken using REDCap, and secure (locked) local storage of original paper-based versions of data collected will occur in accordance with ethically approved procedures for each trial site. Participants will be assigned identification codes on enrolment to the study. These codes will be used during data entry so that data are de-identified during analyses and only aggregated data reported to protect the privacy of participants.

### Statistical methods

The primary analysis will be by intention to treat. Comparison between the intervention and the control groups in the difference from baseline in the passive range of wrist extension (primary outcome) will be presented as the mean difference between the groups and its 95 % confidence interval, obtained using linear regression adjusted for the stratification factors of site and range of passive wrist extension at baseline. The regression model will be fitted using generalised estimating equations (GEE) to allow for the clustering of observations within children for those with both limbs in the study. To explore the effect of the adherence to WHO wearing schedule (i.e. a dose response relationship), a linear regression model will be fitted with compliance to treatment as a predictor and difference in the passive range of wrist extension from baseline to 36 months as the outcome, applied to all study participants. Again this model will be fitted using GEEs to allow for the clustering of limbs within participants. Evidence for an interaction between age and treatment, and between severity (Neurological Hand Deformity Classification) and treatment, will be explored by the inclusion of interaction terms in the linear regression models as well as GEE models. The analyses will be repeated, adjusting for potential confounders including occasions of upper limb BoNT-A injections and frequency of upper limb intervention. Analysis will also be undertaken using the ‘per protocol’ population excluding children who received surgical intervention or casting during the study. All data available from children who are withdrawn from the study prior to study completion will be used for analysis. Imputation of missing data will only be considered in the primary analysis if less than 10–20 % of the primary outcome is missing and will be undertaken throughout multiple imputation models.

Depending on whether data are continuous, categorical or dichotomous, the appropriate generalized linear model will be used to estimate the effect of treatment across the study period on secondary outcomes, again fitted using generalised estimating equations. All analyses will be adjusted for the same stratification factors as for the primary analysis and carried out on intention to treat and per protocol populations.

## Trial governance

A clinical trials agreement is in place between ACU and each trial implementation site that indicates joint intellectual property. A Steering Committee, which includes two parent advisors and all chief investigators, will ensure the study is completed according to the protocol, ethical standards and established timeframes. In addition, the Steering committee will undertake management of the evaluation of the trial and be responsible for establishing a dissemination plan, including peer reviewed publications. Dissemination activities, including attribution of authorship will be undertaken in accordance with the Australian Code for the Responsible Conduct of Research [40]. There are no publication restrictions. The trial Management Committee, based at ACU in Victoria will oversee and manage the day to day operations of the study. State-based advisory groups in Victoria, New South Wales and Western Australia will undertake state-specific implementation. An independent three-person Data Monitoring Committee will review, based on the Damocles Charter, safety, efficacy, participant retention and protocol compliance data and advise regarding protocol variation [11].

## Trial status

Ethical approval has been received from each participating site; study operating procedures and data collection methods have been finalised; and staff recruited and trained in reliable data collection. Recruitment commenced in 2015 and will be ongoing through 2016.

## Discussion/conclusion

Hand dysfunction and deformity are prevalent in CP: 85 % of children have spasticity that impacts upper limb structure and function, ≥62 % have wrist flexion deformities, and early onset is common [41]. Strong positive correlations exist between hand posture and function [9, 41]. Hand orthoses are time-consuming to make and are challenging for families to implement and for children to wear, but if they prevent deformity and improve hand function, they are a vital treatment. This RCT should provide high quality evidence to resolve the long debate about the value of WHO and the specific impact of wrist impairment on activity. In addition, three novel measurement devices will be designed and/or engineered, tested and validated in children within the conduct of this trial: (i) TherApp; (ii) within-orthosis tactile sensors; and (iii) inertial motion sensors. The further application of these devices in a diverse range of clinical and research contexts will constitute a significant intellectual and practical contribution to the health sciences. TherApp has potential for application to support data collection in other health intervention research trials and in clinical practice to support the implementation of interventions and facilitate communication between clients and clinicians. Inertial motion sensors have potential applicability to other interventions focused on outcomes at the body structure and function level of the ICF, such as BoNT-A, and the tactile sensors may also provide data about orthosis fit as well as wearing time, if placed within the orthosis at key points of hand-orthosis contact.

The annual cost of CP in Australia is approximately $1.5 billion (0.14 % of GDP) [42]. Lost wellbeing (as a result of disability and premature death) can be valued at a further $2.4 billion [42]. This research will provide Level II (RCT) evidence [43] to inform clinicians, health services, government funding bodies and parents and carers of children with CP whether the provision of orthoses to prevent upper limb impairment is worth the effort and associated costs. This multicentre RCT along with a companion RCT to be implemented with young children under the age of 3 years will provide high quality evidence of the medium-term effect of rigid upper limb orthoses in children with CP. The second trial aims to determine whether provision of a rigid WHO can prevent the occurrence of contracture and deformity in children aged less than 3 years at time of recruitment. By combining the use of rigid orthoses with usual multidisciplinary therapies, these two trials will investigate a combined intervention more reflective of current best practice than has been previously investigated. The results will provide evidence as to whether the use of rigid upper limb orthoses are needed, or if activity-based therapy alone is sufficient to restore and prevent musculoskeletal impairment in children and adolescents with CP.

## Abbreviations

ACU: Australian Catholic University
BoNT-A: Botulinum toxin-A
CCA: Cost consequence analysis
CEA: Cost effectiveness analysis
CP: Cerebral Palsy
CUA: Cost utility analysis
GEE: General estimating equations
MACS: Manual Ability Classification System
NHMRC: National Health and Medical Research Council, Australia
QALY: Quality adjusted life years
RCT: Randomised controlled trial
SD: Standard deviation
WHO: Wrist hand orthoses
